# Supplementary figures and images for: MEPs elicited by multidirectional rotational-field TMS show marked differences compared to unidirectional Figure-of-8 and H7 coils
Source: PLoS One. 2026 Feb 26;21(2):e0343725. doi: 10.1371/journal.pone.0343725 (PMC12944766; doi:10.1371/journal.pone.0343725)

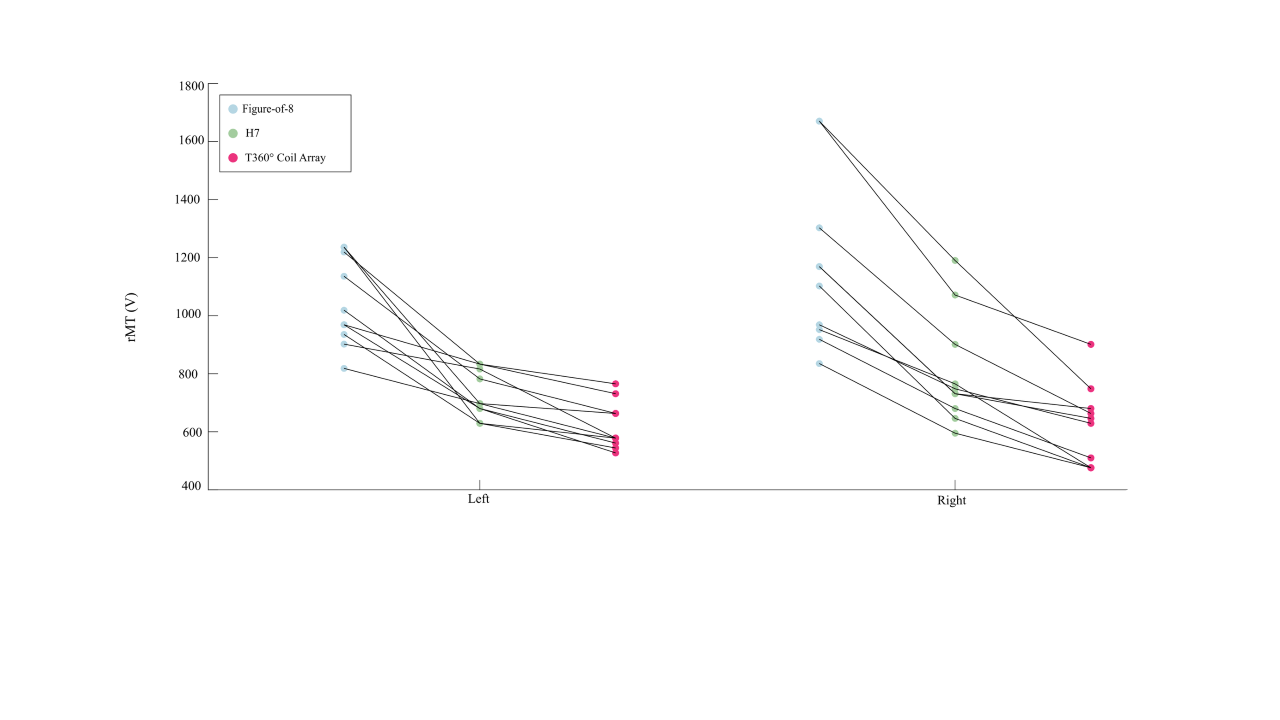

Supplement: S1 Fig — Adjusted resting motor threshold (rMT, volts) upon single-pulse stimulation targeting the hand knob in left and right primary motor cortices, with unidirectional (Figure-of-8 and H7) and multidirectional (rotational-field, T360°) coil configurations and motor evoked potentials (MEPs) derived from the contralateral first dorsal interosseous (FDI) muscle. Each point in the graph represents a single subject, and the black lines join the same subject across the different coils for the same hemisphere (see Results section). (TIF) [file pone.0343725.s001.tif]
