# Supplementary material for: MEPs elicited by multidirectional rotational-field TMS show marked differences compared to unidirectional Figure-of-8 and H7 coils
Source: PLoS One. 2026 Feb 26;21(2):e0343725. doi: 10.1371/journal.pone.0343725 (PMC12944766; doi:10.1371/journal.pone.0343725)
Supplement: Attachment — Submitted filename: Response to the Reviewers _f .docx [file pone.0343725.s004.docx]

23.1.2026

PONE-D-25-48772: *MEPs elicited by multidirectional rotational-field TMS show marked differences compared to unidirectional figure-of-8 and H7 coils.*

**One-by-one responses to the Editor and Reviewers**

We thank the Editor and the three anonymous Reviewers for their most valuable input and constructive comments. We have addressed all the reviewers’ concerns and questions. Additionally, we have followed advice received from reviewers to improve the accuracy of our statistical analysis, as is now reflected in Methods and Results sections. We believe that the modifications introduced to the revised version of the manuscript improved its quality, and we hope they make it suitable for publication in PLoS One. For ease of reference, we bring here the editorial and reviewers' full comments numbered along with our responses. The page and line numbers refer to the revised version of the manuscript, where paragraphs in red ink are those that include phrases that were added, deleted or modified following comments and suggestions made by the reviewers.

**PLOS One editorial requirements:**

When submitting your revision, we need you to address these additional requirements.

**1.**

Please ensure that your manuscript meets PLOS ONE's style requirements, including those for file naming. The PLOS ONE style templates can be found at

<https://journals.plos.org/plosone/s/file?id=wjVg/PLOSOne_formatting_sample_main_body.pdf> and [https://journals.plos.org/plosone/s/file?id=ba62/PLOSOne_formatting_sample_title_authors_affiliations.pdf](https://track.editorialmanager.com/CL0/https:%2F%2Fjournals.plos.org%2Fplosone%2Fs%2Ffile%3Fid=ba62%2FPLOSOne_formatting_sample_title_authors_affiliations.pdf/1/010f019b0b6bdc25-c357c9d2-ed2b-4a19-82b0-6a9fdd961df8-000000/hHtcEGd4KVFBZ5_duSHoqbYZ4J9vXoSm9-es08bBViA=241)

**Response:** The revised version of the manuscript is in accord with PLOS One style requirements.

**2.**

We note that the grant information you provided in the ‘Funding Information’ and ‘Financial Disclosure’ sections do not match. When you resubmit, please ensure that you provide the correct grant numbers for the awards you received for your study in the ‘Funding Information’ section.

**Response:** The accurate information is now inserted in the revised manuscript.

**3.**

Thank you for stating the following financial disclosure: [Funding was received from the LRMC research foundation and from Ariel University to SFT and NS.]. Please state what role the funders took in the study.  If the funders had no role, please state: "The funders had no role in study design, data collection and analysis, decision to publish, or preparation of the manuscript." If this statement is not correct you must amend it as needed. Please include this amended Role of Funder statement in your cover letter; we will change the online submission form on your behalf.

**Response:** The role of funders is now explicitly indicated in the cover letter, as per the above statement (no role in study design, data collection…).

**4.**

Please expand the acronym “LRMC” (as indicated in your financial disclosure) so that it states the name of your funders in full. This information should be included in your cover letter; we will change the online submission form on your behalf.

**Response:** LRMC stands for: “Loewenstein Rehabilitation Medical Center”. The full name appears now in all relevant parts of the manuscript.

**5.**

Thank you for stating the following in the Competing Interests section: [I have read the journal's policy and the authors of this manuscript have the following competing interests: Authors YR and GP are employed by BrainsWay Ltd. All other authors declare no conflict of interest associated with this work. Specifically, no financial or personal relationships with other people or organizations could inappropriately influence or bias this study.].

We note that one or more of the authors are employed by a commercial company: Brainsway Ltd.

1. Please provide an amended Funding Statement declaring this commercial affiliation, as well as a statement regarding the Role of Funders in your study. If the funding organization did not play a role in the study design, data collection and analysis, decision to publish, or preparation of the manuscript and only provided financial support in the form of authors' salaries and/or research materials, please review your statements relating to the author contributions, and ensure you have specifically and accurately indicated the role(s) that these authors had in your study. You can update author roles in the Author Contributions section of the online submission form. Please also include the following statement within your amended Funding Statement. “The funder provided support in the form of salaries for authors [insert relevant initials], but did not have any additional role in the study design, data collection and analysis, decision to publish, or preparation of the manuscript. The specific roles of these authors are articulated in the ‘author contributions’ section.”

If your commercial affiliation did play a role in your study, please state and explain this role within your updated Funding Statement.

**Response:** The required information is now included in the cover letter. BrainsWay Ltd provided support in the form of salaries (to co-authors YR and GP) but had no additional role in the study design, data collection and analysis, decision to publish, or preparation of the manuscript. The specific roles of these authors are articulated in the Author Contributions section, as follows: “The funders had no role in study design, data collection and analysis, decision to publish, or preparation of the manuscript.”

2. Please also provide an updated Competing Interests Statement declaring this commercial affiliation along with any other relevant declarations relating to employment, consultancy, patents, products in development, or marketed products, etc. Within your Competing Interests Statement, please confirm that this commercial affiliation does not alter your adherence to all PLOS ONE policies on sharing data and materials by including the following statement: "This does not alter our adherence to PLOS ONE policies on sharing data and materials.” (as detailed online in our guide for authors <http://journals.plos.org/plosone/s/competing-interests>). If this adherence statement is not accurate and there are restrictions on sharing of data and/or materials, please state these. Please note that we cannot proceed with consideration of your article until this information has been declared. Please include both an updated Funding Statement and Competing Interests Statement in your cover letter. We will change the online submission form on your behalf.

**Response:** Competing Interests: “I have read the journal's policy, and the authors of this manuscript have the following competing interests: Authors YR and GP are employed by BrainsWay Ltd. All other authors declare no conflict of interest associated with this work. Specifically, no financial or personal relationships with other people or organizations could inappropriately influence or bias this study. This does not alter our adherence to PLOS One policies on sharing data and materials."

**6.**

We noted in your submission details that a portion of your manuscript may have been presented or published elsewhere. [It was uploaded as a preprint, DOI: 10.36227/techrxiv.175356536.62169355/v1] Please clarify whether this publication was peer-reviewed and formally published. If this work was previously peer-reviewed and published, in the cover letter please provide the reason that this work does not constitute dual publication and should be included in the current manuscript.

**Response:** We have uploaded an earlier version of this manuscript as a preprint. However, it did not undergo peer review and was not published in a journal.

**7.**

When completing the data availability statement of the submission form, you indicated that you will make your data available on acceptance. We strongly recommend all authors decide on a data sharing plan before acceptance, as the process can be lengthy and hold up publication timelines. Please note that, though access restrictions are acceptable now, your entire data will need to be made freely accessible if your manuscript is accepted for publication. This policy applies to all data except where public deposition would breach compliance with the protocol approved by your research ethics board. If you are unable to adhere to our open data policy, please kindly revise your statement to explain your reasoning and we will seek the editor's input on an exemption. Please be assured that, once you have provided your new statement, the assessment of your exemption will not hold up the peer review process.

**Response:** We assure that all the relevant supporting information files will be available upon acceptance and this is agreed upon all of the authors.

**8.**

We note that Figure 1 in your submission contains copyrighted images. All PLOS content is published under the Creative Commons Attribution License (CC BY 4.0), which means that the manuscript, images, and Supporting Information files will be freely available online, and any third party is permitted to access, download, copy, distribute, and use these materials in any way, even commercially, with proper attribution. For more information, see our copyright guidelines: <http://journals.plos.org/plosone/s/licenses-and-copyright>.

We require you to either (1) present written permission from the copyright holder to publish these figures specifically under the CC BY 4.0 license, or (2) remove the figures from your submission:

1. You may seek permission from the original copyright holder of Figure 1 to publish the content specifically under the CC BY 4.0 license.

We recommend that you contact the original copyright holder with the Content Permission Form (<http://journals.plos.org/plosone/s/file?id=7c09/content-permission-form.pdf>) and the following text:

“I request permission for the open-access journal PLOS ONE to publish XXX under the Creative Commons Attribution License (CCAL) CC BY 4.0 (<http://creativecommons.org/licenses/by/4.0/>). Please be aware that this license allows unrestricted use and distribution, even commercially, by third parties. Please reply and provide explicit written permission to publish XXX under a CC BY license and complete the attached form.” Please upload the completed Content Permission Form or other proof of granted permissions as an "Other" file with your submission.

In the figure caption of the copyrighted figure, please include the following text: “Reprinted from [ref] under a CC BY license, with permission from [name of publisher], original copyright [original copyright year].”

2. If you are unable to obtain permission from the original copyright holder to publish these figures under the CC BY 4.0 license or if the copyright holder’s requirements are incompatible with the CC BY 4.0 license, please either i) remove the figure or ii) supply a replacement figure that complies with the CC BY 4.0 license. Please check copyright information on all replacement figures and update the figure caption with source information. If applicable, please specify in the figure caption text when a figure is similar but not identical to the original image and is therefore for illustrative purposes only.

**Response:** Permission to publish Figure 1A under the Creative Commons Attribution License (CC BY 4.0) has been obtained from the copyright holders via the PLOS Content Permission Form. The completed permission form has been uploaded as an "Other" file with the revised submission, and the figure caption has been updated accordingly.

**9.**

We notice that your supplementary figures are uploaded with the file type “Figure”. Please amend the file type to “Supporting Information”. Please ensure that each Supporting Information file has a legend listed in the manuscript after the references list.

**Response:** All supplementary files were modified as requested.

If the reviewer comments include a recommendation to cite specific previously published works, please review and evaluate these publications to determine whether they are relevant and should be cited. There is no requirement to cite these works unless the editor has indicated otherwise. 

**Reviewer 1.**

This crossover study evaluated the neurophysiological characteristics of a newly developed rotational-field (RF) TMS coil by comparing motor evoked potentials (MEPs) across three coil configurations in 10 healthy adults. The RF coil yielded significantly lower resting motor thresholds and 3-6 fold higher MEP amplitudes relative to conventional figure-of-8 and H7 coils, suggesting enhanced neuronal recruitment through multidirectional stimulation. While these preliminary findings are encouraging for the development of RF coil technology, some suggestions are offered below to optimize the presentation and interpretation of the results.

**1.1.**

Method, Lines 97-99: ”random order (total number of conditions 2x3 separated by 15-30 minutes), spread over one or two testing sessions”. The authors state that the three coil configurations were tested "in a random order" but provide insufficient methodological detail. Please provide a complete description of the randomization procedure, such as the sequence generation method, whether block randomization is used to ensure balanced allocation, how the possible order is distributed among the 10 participants, and other details.

**Response:** We thank the reviewer for this comment. In the current study we applied simple randomization, rather than more complex randomization methods (e.g., block randomization). We now address that in the Methods section, as follows:

- page 6, line 119: “This was carried out via three different coil configurations (Figure-of-8, H7, T360°) in random order (total number of conditions 2x3, separated by 15-30 minutes), spread over one or two testing sessions (see Figure 1A, 1B). Prior to data collection, a series of random numbers was generated using a simple randomization procedure in Microsoft Excel to determine the order of coil and hemisphere conditions. This sequence was manually reviewed to promote a balanced distribution of conditions across participants”.

The reason for choosing simple randomization was practical – our aim was to avoid consistent bias of ordering. As will be noted from the figures, the obtained final cohort was close to be balanced. In addition, due to technical reasons associated with the practical finding of the motor hotspot, stimulation with H7 coil was always followed by the T360° coil array. We acknowledge that this sub-optimal randomization might affect the generalizability of our results, and we address this point in the Discussion section as follows:

- page 17, line 384: “To minimize potential systematic bias from time-dependent effects, the order of experimental conditions was randomized. Although we aimed to counter-balance conditions, the final cohort suffered from minor imbalances (Figure 1B). In addition, due to technical reasons associated with the practical finding of the motor spot, stimulation with H7 coil was always followed by the T360° coil configuration. These imbalances could affect the generalizability of our results.”

**1.2.**

Result, Lines 176-177: "Cases where stimulation intensities differed by more than 5% of the MSO were eliminated from analysis, thus yielding 281 EMG traces out of 1881 valid EMG traces". The exclusion of 85% of valid EMG traces due to a 5% MSO difference threshold is problematic and unnecessarily reduces statistical power. The current method risks introducing selection bias, which could be more serious than the small intensity differences it attempts to control. Please include all 1881 valid traces using mixed-effects models with stimulation intensity as a covariate, or perform a sensitivity analysis using different exclusion thresholds (5%, 10%, 15% MSO) to demonstrate robustness**.**

**Response:** We thank the reviewer for this most valuable comment and suggestion. In the original version of the manuscript, our analysis was indeed restricted to a small subset of the data - specifically, MEP traces recorded at 110% of each coil-, side-, and subject‑specific rMT. We agree that limiting the analysis to this narrow intensity window could introduce selection bias and does not fully exploit the richness of the dataset. Following the reviewer’s advice we have revised our approach and now analyze all 1881 valid EMG traces. Because MEP amplitudes follow a sigmoidal (logistic) relationship with stimulation intensity, we implemented a Bayesian hierarchical model that incorporates stimulation intensity directly as a predictor. This modeling framework is well suited for datasets that are small, noisy, and structurally complex, such as ours. Implementing this revised analysis required corresponding updates throughout the Methods, Results, and Discussion sections. We kindly refer the reviewer to the revised manuscript for checking the rather extensive changes made following this suggestion. The relevant parts are easily identified within paragraphs in red ink (paragraphs that include phrases modified in the revised version of the manuscript). Specifically, we refer the reviewer to the paragraphs starting in the following places: page 9, line 196; page 11, line 250; page 13, line 301.

**1.3.**

Discussion, Lines 244-247: "We expect that this technique could provide beneficial and effective treatment by significantly boosting their potential for upper limb recovery". The authors' strong prediction of treatment outcomes for stroke patients based solely on neurophysiological findings from only 10 healthy adults is clearly overly optimistic. In fact, no patient data are presented to support these clinical claims. The use of definitive language such as "significantly boosting" and "beneficial and effective treatment" is inappropriate for preliminary healthy subject data. Please revise "ALL" relevant content in the discussion section.

**Response:** We thank the reviewer for pointing out this. In response, we have amended the tone of relevant sentences to reflect a more modest standpoint, acknowledging that our data collection involved only 10 healthy participants and that this constitutes a preliminary study. We have done our best to make sure that our tone is less definitive across all of the relevant text. For example:

- page 16, line 358: “…given the broader spatial extent of activation by H and T360° coil configurations, adjacent cortical regions (e.g., premotor and supplementary motor cortices) might be activated. The functional and behavioral implications of such off-target activation remain poorly understood and warrant further investigation. Increases in MEPs should be interpreted cautiously and not taken as evidence of activation supporting improved motor function (i.e., superior corticospinal recruitment leading to enhanced treatment response).[35]”
- page 16, line 368: “…the large majority of studies that have applied TMS to enhance motor recovery post stroke used the Figure-of-8 coil type [4]. However, significant response variability and uncertainty about long-term efficacy have led to recent doubts [30] and have stressed the need for novel approaches. The capacity of rfTMS to recruit larger neuronal populations in multiple orientations at the stimulation site, with lower stimulator outputs [6,12], may advance the field of therapeutic brain stimulation for enhancement of motor recovery. Following a stroke, reorganization of motor networks involves multidirectional axonal sprouting and new synapse formation [37]. The increased ability of rfTMS to stimulate neurons in various orientations may thus have a positive impact on the likelihood of benefit from TMS-based rehabilitation therapies.”
- page 16, line 378: “While this study introduces novel options for using rfTMS, it also has several limitations. The most notable are the small sample size and the wide age range of participants. The broad age range…” (analysis of limitations of the study).
- Page 17, line 396: “The current study represents the initial phase of an ongoing project, where in the near future, therapeutic rTMS will be applied via the rotational-field coil configuration, in stroke patients with severe hemiparesis and chronic non-functional upper limb, for the first time. We hope the advantages demonstrated in this preliminary study will prove beneficial for patients.”

**Reviewer 2.**

This study compares three different TMS coils (Figure-of-8, H7, and RF). The authors report that the RF coil yields a lower resting motor threshold and elicits larger motor evoked potentials compared to unidirectional coils. While the findings hold promise for future applications in stroke rehabilitation, the study is limited by a small sample size. I have the following questions and suggestions regarding this study.

**2.1.**

The participants in this study span a broad age range (30-73 years). Given that age is known to significantly influence both resting motor threshold (rMT) and motor evoked potentials (MEPs), the current manuscript does not explain how this potential confounding factor was controlled for in the analysis. It is suggested that the authors add an explanation of how age was handled or explicitly discuss this in the Limitations section.

**Response:** We understand and value the reviewer's concern regarding this significant issue. Our recruitment strategy was purposely designed to prioritize variety corresponding to the stroke population. This approach may have mitigated age-related trends in rMT within our sample, although such trends are frequently documented in past studies. A substantial cohort of stroke patients reported in a previous study by our research group serves as a reference for the typical age distribution of the stroke population undergoing rehabilitation (Frenkel-Toledo et al., PLOS ONE, 2019, e0219738), and these data informed the recruitment strategy for the current study to reflect the clinical stroke population better. Age was not included as an explicit covariate in the statistical model. Instead, our Bayesian hierarchical framework added to the revised version following comment and suggestion received from Reviewer 1 (see please our response to his/her comment #1.2) incorporates subject‑level random effects, which help account for inter‑individual variability, including variability potentially associated with age. Given the modest sample size and the wide but continuous age distribution, introducing age groups or additional covariates would have been statistically impractical and risked overfitting. Nevertheless, we agree that age may influence corticospinal excitability, and we now explicitly acknowledge this as a limitation in the revised manuscript, as follows:

- page 16, line 378: “While this study introduces novel options for using rfTMS, it also has several limitations. The most notable are the small sample size and the wide age range of participants. The broad age range was intentional, aiming to reflect the typical clinical population. Age‑related variability was partially accommodated in the I/O curve modelling through the inclusion of subject‑level random effects. Moreover, the use of…”

**2.2.**

While the experimental design employs single-pulse TMS, specific parameters are missing, such as the inter-pulse interval. It is suggested that the authors provide these details. Additionally, the legend for Figure 1 labels the image as “Study design,” yet the figure content only displays the shapes and setup of the different coils. Please verify whether the figure content or the legend requires modification to accurately reflect the content.

**Response:** We thank the reviewer for drawing our attention to this issue. The inter-pulse interval in the single-pulse TMS protocol was set between 5 to 7 seconds. This detail has now been incorporated into the Methods section. Additionally, we provide now more information in the Methods as also requested by the third reviewer. We added another figure to the study design (Figure 1B) and renamed the original figure (Figure 1A in the revised version) "Coil geometry" as recommended. The manuscript was revised accordingly:

- page 7, line 149: “All stimulations were delivered with a fixed inter-stimulus interval (ISI) between 5 and 7 seconds [21,22].”
- page 7, line 154: “Figure 1 provides a schematic illustration of coil geometry (Figure 1A) and experimental workflow (Figure 1B).”

**2.3.**

The interval between each condition was 15–30 minutes; is this sufficient to eliminate the effects of the previous stimulation? Furthermore, given the prolonged duration of the experiment, could this reduce the subjects' corticospinal excitability and introduce experimental biases?

**Response:** We thank the reviewer for this comment. Indeed, several authors argue for a benefit of using longer inter-stimulus intervals (Pellicciari et al., Clinical Neurophysiology, 2016, pp. 621–628). However, ISIs of 5-10 seconds are commonly reported in the literature and here we followed this practice. By minimizing the overall assessment time, we enabled comparative testing of one hemisphere, with all three coil configurations, in a relatively short time, in one and the same day, thus reducing fatigue effects. This practice mitigated inter-coil variability attributed to stimulation in different days. We also tried to minimize possible related bias by counter-balanced ordering of the coils (Figure 1B). Nevertheless, we now explicitly acknowledge in the revised manuscript that some degree of temporal drift in excitability is possible and represents a limitation of the study, as follows:

- page 17, line 389: The inter-stimulus interval (ISI) used in this study was of 5–7 seconds, as done in previous studies [21,22]. While this ISI was important for minimizing variance attributed to subjects’ fatigue, it might have introduced a bias due to cumulative neural effects.

**2.4.**

Regarding TMS coil positioning, this study relied solely on traditional landmarks. In the absence of MRI-guided neuronavigation, is this sufficient to accurately target the primary motor (M1) region? Furthermore, how was it confirmed that the hot spot for the H7 and RF coils precisely corresponds to the M1 region?

**Response:** We thank the reviewer for this comment. While acknowledging the advantages of MRI-guided neuronavigation, the present study aimed to compare resting motor threshold and MEP I/O curves across different TMS coils rather than to conduct precise anatomical mapping of the primary motor cortex (M1). The search for the M1 location, whether using a focal Figure-of-8 coil or a combination of deep H7 or T360^0^ (rfTMS) coils, was refined through electromyography (EMG)-based motor hotspot localization. Once the hotspot was identified, we marked this area on a cap for the Figure-of-8 coil. Later, the same cap was marked with a grid for the H7 and rfTMS coils. In both cases, the scalp position was maintained. For the H7 and rfTMS coils, the helmet was secured to ensure consistent stimulation of the same site across measurements. Moreover, the same operator (first author OWBS) performed all manual procedures and was directly holding the coil in all conditions, thus reducing possible effects of inter-operator variability. In the absence of MRI-guided neuronavigation, exact anatomical coordinates of the stimulation site or its precise relationship to individual cortical sulci cannot be determined. However, because the same EMG-defined motor hotspot was used for all three coils within each participant, any residual spatial uncertainty is shared across conditions and is unlikely to bias the within-subject comparison between coils. The manuscript was revised accordingly to reflect this point:

- page 19, line 392: “Lastly, although neuronavigation was not used in the present study, the hotspot location was verified using EMG for all three coils in each participant. Hence, any residual spatial uncertainty was shared across conditions and was unlikely to bias the within-subject comparison between coils.”

**2.5.**

In the Results section, the MEPs elicited by the H7 coil show significant asymmetry (0.187 mV for the right hemisphere, and 0.47 mV for the left) compared to the Figure-of-8 and RF coils. It is suggested that the authors explain the potential physiological or technical factors contributing to this asymmetry in the manuscript.

**Response:** We thank the reviewer for drawing attention to this point. The asymmetry noted in the earlier version of the manuscript arose from the restricted analysis of peak‑to‑peak MEP amplitudes at 110% rMT. In the revised analysis - based on full I/O curve modelling - we again observe a higher maximal amplitude (log A parameter) for the left hemisphere when using the H7 coil, which is consistent with the reviewer’s observation. As noted in the manuscript, this hemispheric difference has not been reported previously and warrants further investigation. The fitted I/O curves suggest that the H7 coil may engage a larger cortical volume than the Figure‑of‑8 coil. If the dominant‑hemisphere M1 contains a denser or more excitable neuronal population, a coil with greater depth and spread could amplify this asymmetry. The T360° coil configuration also stimulates larger brain volumes compared to the Figure-of-8 coil, potentially approaching a ceiling effect that reduces observable inter‑hemispheric differences. Because these inter‑hemispheric effects were not consistent across coils, their interpretation remains speculative without targeted experimentation. Therefore, we chose not to elaborate on them in the revised manuscript, which focuses on inter‑coil comparisons. We view this as an interesting direction for future, dedicated work.

**2.6.**

Additionally, the legend for Figure S1 mentions that 3 subjects were excluded from the right hemisphere analysis and 1 subject from the left hemisphere analysis; is this data exclusion related to the asymmetry in the H7 coil MEP results? It is recommended that the exclusion of these subjects and the reasons for doing so be explicitly mentioned in the manuscript.

**Response:** We thank the reviewer for raising this point. In the earlier analysis, three subjects were excluded from the right‑hemisphere dataset: one due to excessive noise caused by a hardware malfunction that prevented reliable peak‑to‑peak MEP measurement, and two because their resting motor thresholds (rMTs) were so high that MEPs could not be obtained at 110% rMT. In the revised analysis, which incorporates all available stimulation intensities rather than restricting the dataset to 110% rMT, the two subjects with high rMTs are now included. Importantly, the Bayesian hierarchical model used in the revised version of the manuscript incorporates a truncation component that explicitly accounts for missing data arising when rMT exceeds the maximal stimulator output. This allows the model to estimate I/O curve parameters while reducing potential bias associated with structurally missing observations.

__________________________________________________________________

**Reviewer #3**

The manuscript addressed an important methodological comparison of coil configurations and introduces a multidirectional rfTMS system. The results are promising, but several conceptual, methodological, and reporting issues must be clarified before publication.

**Major comments:**
**3.1.**

The Introduction lacks a clearly articulated rationale connecting MEP properties, coil physics, and motor rehabilitation relevance. The authors should specify hypotheses regarding how multidirectional stimulation may improve recruitment or prognostic interpretation. For example, clearly define why MEPs matter for diagnosis/prognosis, explain how coil configuration might influence MEP detectability.

**Response:** We thank the reviewer for pointing out this matter. We have included expanded explanations of these issues in the revised version of the manuscript, in both introduction and discussion sections of the text, as follows:

- page 4, line 66: “Damage to the corticospinal tract (CST) is the most important determinant of upper-limb function post stroke, with severe damage predicting low likelihood of late recovery. Evaluation of MEPs is widely used to assess the residual capacity for neural transmission in the CST. MEPs are therefore commonly used as key biomarkers to predict stroke recovery and to stratify patients for personalized rehabilitation approaches [13,14], particularly in the context of interventions incorporating non-invasive brain stimulation [4]. However, MEP properties are influenced not only by CST integrity but also by stimulation-related factors (e.g., inter-trial interval, stimulation intensity, coil orientation relative to the hotspot, the spatial spread of the electric field induced by TMS), and by individual differences in neuroanatomy and motor-system responsiveness. Additionally, MEP amplitudes show noticeable trial-to-trial variability when recorded at a single stimulation intensity. Thus, previous researchers have proposed that assessing MEPs across a range of stimulus intensities using input–output (I/O) curves can elucidate cortical excitability more than single-intensity measurements [15–17]. The sigmoidal I/O relationship measures recruitment gain, maximum corticospinal output, and the dynamic range of excitability at different intensities. It may also mitigate sensitivity to temporary fluctuations in brain state and strengthen test-retest reliability.

With conventional unidirectional Figure-of-8 TMS, neuronal recruitment is highly sensitive to the orientation of the induced electric field relative to cortical geometry, potentially limiting activation of corticospinal neurons in orientations that differ from that of the induced electric field [8]. Multidirectional or rotational-field stimulation may reduce this orientation dependence by capturing spatially distinct neuronal populations, thereby increasing the likelihood and robustness of measurable MEPs [11]. In this context, understanding how coil configuration influences MEP properties is critical to interpreting MEP-based biomarkers in stroke rehabilitation.

TMS has been experimentally applied in stroke rehabilitation for many years, with the aim of enhancing adaptive neuroplasticity and improving functional outcomes. In the majority of cases, stimulation is delivered via the Figure-of-8 coil. Response variability is large, and randomized control studies yield mixed results [4]. Enhanced robustness of neuroplastic effects through deeper and wider neuronal recruitment may pose a significant advantage for TMS-based therapies, yet exacerbation of side effects and the potential activation of adjacent inhibitory networks is a matter of concern [12].

Selection of the optimal stimulation target for an individual hemiparetic stroke patient is, to date, an unsolved issue. Clinical algorithms, like the widely used PREP2 [18] recommend stimulating the lesioned/non-lesioned hemisphere on the basis of the status of motor evoked potentials (MEP+/MEP-, respectively) plus other clinical biomarkers. Yet, whether this binary approach truly optimizes prognostication and clinical decision making was recently questioned [14], and alternative approaches were suggested [19]. Given the importance of MEP status in clinical rehabilitation practice, the extent to which MEP properties depend on TMS coil configuration was investigated. Specifically, properties of MEPs elicited from the contralateral first-dorsal interosseous (FDI) muscle were assessed. A comparison was made between udTMS coils (Figure-of-8 and H7) and the multi-directional dual H rotational field TMS coil (rfTMS, also termed T360°).

- page 14, line 313: The broad, multidirectional stimulation of the T360° coil configuration may be advantageous for therapeutic neuromodulation, particularly in post-stroke populations in which networks are reorganized, and widespread activation enhances plasticity. With these advantages, rfTMS may indicate the existence of residual corticospinal neural transmission in cases of severe stroke where contralateral motor response cannot be elicited by unidirectional TMS. These advantages of rfTMS may also lead to improved outcomes in rehabilitation treatments employing rTMS, potentially decreasing the high response variability currently observed in such treatments, thus lowering the doubts about their effectiveness [30]. Additionally, a lower rMT could lead to reduced patient discomfort during TMS sessions and mitigate the risk of adverse side effects associated with higher intensities of stimulation, which is a noted concern with elevated stimulation levels [7,8].”
- page 16, line 365: “A recent study has indicated the potential benefits of repetitive TMS applied via the T360° coil configuration for patients with major depression [36]. Today, coils of the H type, either in single or dual (rfTMS) coil configuration, are in use mainly for psychiatric disorders. In contrast, the large majority of studies that have applied TMS to enhance motor recovery post stroke used the Figure-of-8 coil type [4]. However, significant response variability and uncertainty about long-term efficacy have led to recent doubts [30] and have stressed the need for novel approaches. The capacity of rfTMS to recruit larger neuronal populations in multiple orientations at the stimulation site, with lower stimulator outputs [6,12], may advance the field of therapeutic brain stimulation for enhancement of motor recovery. Following a stroke, reorganization of motor networks involves multidirectional axonal sprouting and new synapse formation [37]. The increased ability of rfTMS to stimulate neurons in various orientations may thus have a positive impact on the likelihood of benefit from TMS-based rehabilitation therapies.”

**3.2.**

The procedural description requires greater specificity. For example, the manuscript does not state how many trials were delivered for each coil and hemisphere, nor does it clarify whether stimulations were randomized, blocked, or interleaved, or whether the order of coil configurations was counterbalanced across participants. The inter-trial interval (ITI) was also not reported. In addition, the description of hotspot determination was too brief; the authors should specify the method used to locate the hotspot (e.g., grid search procedure), the number of stimulations used to confirm it, whether neuronavigation was implemented, and whether the hotspot location differed across coil types. Similarly, the criteria used to define the resting motor threshold (rMT) were not described. It should be stated explicitly whether the standard definition (i.e., at least 5 out of 10 MEPs exceeding 50 µV) was used. Clarifying these points was essential for ensuring methodological transparency and replicability. In addition, the experimental procedure should be illustrated in figure 1.

**Response:** We thank the reviewer for drawing our attention to this missing information. We have expanded the Methods section to clarify the experimental procedure, including hotspot localization, stimulation order, interstimulus interval, and determination of resting motor threshold. These details are now described explicitly in the revised manuscript and illustrated in Figure 1B.

- page 7, line 118: “Procedure: A crossover design was used, applying single-pulse TMS (spTMS) over the left- and right-hand regions of the primary motor cortex. This was carried out via three different coil configurations (Figure-of-8, H7, T360°) in random order (total number of conditions 2x3, separated by 15-30 minutes), spread over one or two testing sessions (see Figure 1A, 1B). Prior to data collection, a series of random numbers was generated using a simple randomization procedure in Microsoft Excel to determine the order of coil and hemisphere conditions. This sequence was manually reviewed to promote a balanced distribution of conditions across participants.

Hotspot localization and resting motor threshold (rMT) determination were conducted separately for each coil type and for each tested hemisphere. During this process, a systematic positional search was performed by incrementally moving the coil in anterior–posterior (1 cm) and medial–lateral (0.5 cm) directions while maintaining a constant orientation. Hotspot localization was performed independently for the Figure-of-8 coil and the BrainsWay coils. At each tested position, at least 2–3 single TMS pulses were delivered before determining whether the location elicited a reliable MEP. The motor hotspot was defined as the scalp location that consistently produced the largest and most stable MEP amplitudes at a fixed stimulator output. Neuro-navigation was not used; instead, standardized anatomical landmarks and cap-based grid coordinates were applied.

Resting motor threshold (rMT) was determined using an adaptive threshold-hunting procedure implemented via the Motor Threshold Assessment Tool (MTAT 2.0; Awiszus & Borckardt), [20] rather than the conventional ‘5-out-of-10’ method. The initial stimulator intensity was set based on the output used during hotspot localization. The threshold was defined according to the predefined MEP amplitude criterion (100 µV), as detailed above, in accordance with the adaptive algorithm's convergence rules. This method has been shown to provide accurate and reliable rMT estimates while reducing the total number of TMS pulses required.

The order of coil configurations and the hemisphere side were counterbalanced across participants and were determined using simple random number generation. In addition to the single TMS pulses required for determining the hot spot and the rMT, five stimuli at seven stimulation intensities were delivered to the FDI hotspot. Stimulation intensities were fixed and ranged from 90% to 150% of the specific rMT for the condition (subject, coil and side) in 10% increments. All stimulations were delivered with a fixed inter-stimulus interval (ISI) between 5 and 7 seconds [21,22].

Overall, the study employed a randomized cross-over design, such that all participants were exposed to all coil configurations while serving as their own control. This counterbalancing procedure was implemented to minimize carryover effects and promote randomness across coil type and hemisphere. Figure 1 provides a schematic illustration of coil geometry (Figure 1A) and experimental workflow (Figure 1B).

**3.3**

Regarding safety and tolerability, the manuscript asserted that a lower rMT may reduce the risk of adverse effects, yet it does not report whether any side effects or discomfort actually occurred during the study. A brief section should be added describing whether participants experienced pain, muscle twitches outside the targeted FDI muscle, headaches, or any other sensations during stimulation. It would also be important to note whether the subjective experience of stimulation intensity differed across coil types. Including these details would strengthen the manuscript by providing a more complete account of participant safety and tolerability.

**Response:** We thank the reviewer for pointing out this important matter. We have added the following information to the manuscript.

- page 14, line 292: “All subjects tolerated the study well, and there were no significant adverse events. However, physical reactions to TMS pulses were often more noticeable for the H7 and rfTMS coils compared with the Figure-8 coil, particularly in the form of blinking and facial twitching. This was expected given the wider spread of the electric field of deep TMS coils. One subject reported temporary tooth pain during the pulses, which quickly disappeared, and another subject reported a headache the day after.”

**3.4.**

The Discussion acknowledged that H-coils stimulate deeper and broader cortical areas. However, the implications of this reduced focality for motor specificity remain insufficiently addressed. Existing electric-field models, including the simulations presented in Roth et al. (2020), indicated that rfTMS produced a substantially deeper and wider field compared with both figure-of-8 and single H-coils. Because the rotational field covered a large cortical volume and stimulated axons across multiple orientations, the authors should clarify how such broad activation aligned with the goal of targeting specific motor representations in M1. A more detailed discussion was warranted on several conceptual points. First, how “focal” was the RF coil according to current modeling work, and could the multidirectional fields disrupt somatotopic specificity within the motor cortex? Second, given the coil’s broader spread, was there a realistic risk of activating adjacent or unintended regions such as the premotor cortex or supplementary motor area? Including or citing electric-field simulations would significantly strengthen the readers’ understanding of these issues. Moreover, the authors’ interpretation of larger MEPs as indicative of greater corticospinal recruitment should be balanced with the recognition that rfTMS stimulated a larger tissue volume, which may amplify MEPs simply through increased spatial coverage rather than improved corticospinal integrity. In other words, a larger MEP does not necessarily translate to better function or superior treatment response. This distinction is important both scientifically and clinically.

**Response:** We thank the reviewer for pointing out these important issues. We have added an expanded explanation of these issues in the revised version of the manuscript, as follows:

- Page 15, line 347: “For diagnostic purposes, such as evaluating corticospinal tract integrity, establishing motor thresholds, or generating somatotopic MEP maps, a more focal and unidirectional stimulation is desirable. One option is the conventional Figure-of-8 coil due to its focality. Similarly, when operated at the threshold for motor activation, H-coils such as the H7 can produce focal stimulation at the motor cortex representation of specific movements, without activating additional movements. Moreover, by operation of the two orthogonal coils of the T360° coil configuration simultaneously (and not with a lag, as done to achieve a rotating field), and calibration of the relative amplitudes of the two channels, one can probe all the possible orientations without moving the coils. This may be a powerful tool for the investigation of brain networks and corticospinal tracts, both at rest and during a task, with comparable probing of all orientations. Yet, the possibility of stimulating areas beyond the intended target region cannot be ruled out, and given the broader spatial extent of activation by H and T360° coil configurations, adjacent cortical regions (e.g., premotor and supplementary motor cortices) might be activated. The functional and behavioral implications of such off-target activation remain poorly understood and warrant further investigation. Increases in MEPs should be interpreted cautiously and not taken as evidence of activation supporting improved motor function (i.e., superior corticospinal recruitment leading to enhanced treatment response).[35]”

**3.5.**

Following the context drafted in comment 4, it may be valuable for the authors to differentiate between the potential uses of rfTMS in rehabilitation versus diagnostic assessment. The broad, multidirectional stimulation of the RF coil could be advantageous for therapeutic neuromodulation, particularly in post-stroke populations where networks are reorganized and where widespread activation may enhance plasticity or reveal residual pathways not captured by conventional TMS. However, for diagnostic purposes, such as evaluating corticospinal tract integrity, establishing motor thresholds, or generating somatotopic MEP maps, the conventional figure-of-8 coil might be superior due to its focality and interpretability. If the authors agree with this distinction, incorporating it into the Discussion or Limitations section would improve conceptual clarity and strengthen the manuscript’s translational relevance.

**Response:** We thank the reviewer for this valuable comment. These points were elaborated upon and clarified in the discussion section, as follows:

- page 17, line 347: “For diagnostic purposes, such as evaluating corticospinal tract integrity, establishing motor thresholds, or generating somatotopic MEP maps, a more focal and unidirectional stimulation is desirable. One option is the conventional Figure-of-8 coil due to its focality. Similarly, when operated at the threshold for motor activation, H-coils such as the H7 can produce focal stimulation at the motor cortex representation of specific movements, without activating additional movements. Moreover, by operation of the two orthogonal coils of the T360° coil configuration simultaneously (and not with a lag, as done to achieve a rotating field), and calibration of the relative amplitudes of the two channels, one can probe all the possible orientations without moving the coils. This may be a powerful tool for the investigation of brain networks and corticospinal tracts, both at rest and during a task, with comparable probing of all orientations.”

Minor comments:

**3.6.**

The manuscript used first-person pronouns, which was not typical for academic writing. Replacing it with passive voice or a third-person description would be more formal.

**Response:** the manuscript was revised to passive voice as requested.

**3.7.**

The reference format was inconsistent. The mixed use of APA-like elements, URLs, and irregular punctuation made the reference list appear informal. A full revision of the references to meet journal standards is necessary.

**Response:** We appreciate the reviewer's feedback and have revised the references to meet the journal's standards.

**3.8**

The terminology used in the manuscript would be better to keep consistent, e.g., RF, T360o, and rfTMS, and ensure all abbreviations are defined upon first use.

**Response:** We thank the reviewer for the comment. We have addressed this issue in the revised version of the manuscript. For example, we now refer to the coil which produces rotational-field TMS as “T360°” to indicate the device and rfTMS to indicate the principle of operation of this coil configuration.
